# Mastering of NIL Stamps with Undercut T-Shaped Features from Single Layer to Multilayer Stamps

**DOI:** 10.3390/nano11040956

**Published:** 2021-04-09

**Authors:** Philipp Taus, Adrian Prinz, Heinz D. Wanzenboeck, Patrick Schuller, Anton Tsenov, Markus Schinnerl, Mostafa M. Shawrav, Michael Haslinger, Michael Muehlberger

**Affiliations:** 1 TU Wien, Institute for Solid State Electronics, 1040 Vienna, Austria; philipp.taus@tuwien.ac.at (P.T.); patrick.schuller@tuwien.ac.at (P.S.); anton.tsenov@tuwien.ac.at (A.T.); markus.schinnerl@tuwien.ac.at (M.S.); 2 Stratec Consumables GmbH, 5081 Anif, Austria; a.prinz@stratec.com; 3 ams AG, Tobelbader Strasse 30, 8141 Premstaetten, Austria; mostafa.shawrav@gmail.com; 4 PROFACTOR GmbH, 4407 Steyr, Austria; michael.haslinger@profactor.at (M.H.); michael.muehlberger@profactor.at (M.M.)

**Keywords:** nanoimprint lithography (NIL), undercut features, master, Blu-Ray patterning, reactive ion etching

## Abstract

Biomimetic structures such as structural colors demand a fabrication technology of complex three-dimensional nanostructures on large areas. Nanoimprint lithography (NIL) is capable of large area replication of three-dimensional structures, but the master stamp fabrication is often a bottleneck. We have demonstrated different approaches allowing for the generation of sophisticated undercut T-shaped masters for NIL replication. With a layer-stack of phase transition material (PTM) on poly-Si, we have demonstrated the successful fabrication of a single layer undercut T-shaped structure. With a multilayer-stack of silicon oxide on silicon, we have shown the successful fabrication of a multilayer undercut T-shaped structures. For patterning optical lithography, electron beam lithography and nanoimprint lithography have been compared and have yielded structures from 10 µm down to 300 nm. The multilayer undercut T-shaped structures closely resemble the geometry of the surface of a Morpho butterfly, and may be used in future to replicate structural colors on artificial surfaces.

## 1. Introduction

In biology, surfaces with three-dimensional nanostructures are responsible for outstanding physicochemical properties of the surface. The anti-wetting surface of the lotus flower, the low friction surface of shark skin and the high sticking capability of Gecko feet are well-described. Another exceptional example is structural color: Not chemical pigmentation, but a complex structured multilayered nanostructure, is the cause of the brilliant blue wing colors in some of the species of morpho butterflies and the golden color of the beetle *Chrysina aurigans* [1,2].

Imitating nature’s nanostructured surfaces by engineering methods promises to gain the same spectacular properties on human-made products. The biomimetic replication of a lotus-like surface for anti-wetting features has been described [3]. A wide range of lithographic methods has been successfully applied for three-dimensionally structured surfaces [4,5,6,7]. However, many methods can only generate these biomimetic structures on a small area. The challenge is the fabrication on a larger surface area—here nanoimprint lithography has a lot of potential [8].

Nanoimprint lithography (NIL) is a cost-effective method to replicate nanostructures on large areas [9,10]. A nanostructured stamp is used to mechanically emboss a polymer layer on a substrate [11]. Such three-dimensionally structured surfaces are interesting for microfluidic channels [12,13], superhydrophobic surfaces [14], and optical applications [15,16].

Also undercut structures [11,17] and hierarchical structures [18] can be fabricated. With an imprint stepper or with roll-to-roll NIL also large areas can be patterned three-dimensionally. For fabrication of three-dimensional structures hybrid structuring processes combining NIL and electron beam lithography [19,20] or NIL and photolithography [21], as well as multilayer nanoimprinting [22] to create hierarchical stamp masters for optical micro- and nanostructures have been recently demonstrated successfully. Even imprinting of complex three-dimensional nanomolds [23] was successfully demonstrated.

In contrast to the layer by layer fabrication with conventional multilevel lithography, the NIL fabrication allows to directly replicate a complete 3D structure in a single patterning step [24]. Master stamps are a critical component in the nanoimprint process. While initially these masters were directly used as stamps, now it is more common to replicate a working stamp from the original master and then use this working stamp for the actual nanoimprint process of the final product.

Often the masters are made from silicon wafers using well-established silicon technology, like electron beam lithography or interference lithography combined with reactive ion etching [10], but also biological samples can be used to directly replicate bionic structures [4,25]. The fabrication of three-dimensional master stamps has also been achieved by focused ion beam related methods [26,27] or by grey scale lithography [28].

The replication of nanostructures in the nanoimprint process is commonly limited to draft angles equal or smaller than 90°, but recently it was also shown that undercut structures as well as nano-cavities could be replicated when the right materials for the stamp and imprint material are chosen [11,29]. The process parameters of the replication—especially of the demolding step—are critical for freeform microstructures [30]. Also, innovative alternatives such as shape memory polymers have been reported [31,32]. Not only is the replication process a challenge, but also the fabrication of the master itself.

The objective of this work is to fabricate the multilevel nanostructured surface resembling geometry of a Morpho menelaus wing. The challenge is that multilayer undercut features have to be fabricated. Such structures are not only beneficial for biomimetic structures, a similar requirement also exists for vertical-channel solid-state memory [33,34]. This paper describes and compares the fabrication of (A) single-layer undercut T-structures using PTM-layers (phase transition material) on silicon as well as (B) multi-layer undercut T-structures of a Si-SiO_2_-multi-stack (Table 1). The PTM-layers on silicon were patterned mask-less by Blu-Ray laser writing [35]. The Si-SiO_2_-multi-stack was etched by RIE using gold hardmasks patterned by three different lithography methods—optical lithography, electron beam lithography, and NIL.

## 2. Materials and Methods

As substrate for the master, Si (100) wafers were used. These wafers were used to fabricate two types of masters: (i) single-layer undercut structures with a T-shaped cross-section and (ii) multilayer undercut structures with a T-on-top-of-T-shaped cross-section. To accomplish these structures, a multilayer stack of different materials was deposited by sputter deposition. The materials deposited were silicon, silicon oxide and phase transition material (PTM) [36]. The lateral structure was defined by lithographic patterning of the top layer as described in the following sections:

### 2.1. Single Layer Undercut Masters

**Figure 1:**
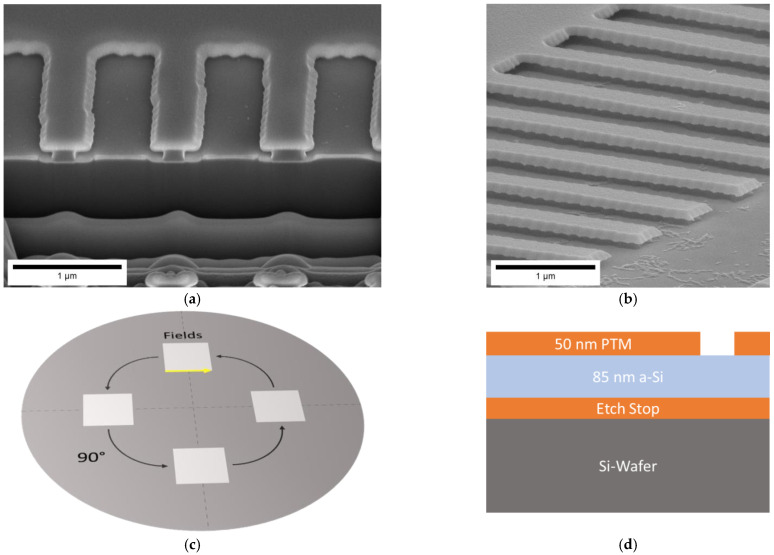
Single layer undercut master. (**a**) SEM image of a FIB-cross-section showing the underetched geometry (**b**) Tilted view SEM image of the single-layer underetched structures. The PTM top layer forms the overhanging ledge on top of the underetched Si base. (**c**) Optical image of wafer is indicating the Blu-Ray mastering of the top PTM layer (**d**) Schematic cross-section through the layer stack.

To prepare the single-layer T-shaped masters (see Figure 1) a layer stack of PTM (MoWO_x_), amorphous silicon (a-Si) and PTM were deposited on a 200 mm Si wafer by sputter deposition. The top PTM layer was 50 nm thick and was processed to act as a hard mask. The bottom PTM layer served as an etch-stop layer. For patterning of the top layer, the PTR-3000 phase transition mastering system (Sony Disc Technology Inc., Tokyo, Japan) was used. The PTR 3000 system was developed for Blu-Ray mastering and uses a 405 nm diode laser for exposure [37]. Blu-Ray mastering allows high-speed large area writing with resolutions down to 130 nm [35]. The phase transition material behaves comparable to a positive photoresist and changes from amorphous to polycrystalline upon laser irradiation. Compared to regular Blu-Ray mastering, this process used constant angular velocity writing with a rotation velocity of 450 rpm and a 7.75 ns clock cycle, which results in pit sizes of 7.3 nm at 20 mm radius and 14.6 nm at 40 mm. The pit-density was increased from inner tracks towards outer tracks (trackpitch 40 nm) in software, to achieve a homogenous exposure. The exposed area can be developed using a 2.38% TMAH wet chemistry for 2000 s. With the top PTM layer as hard mask, the underlying layer of amorphous Si was wet etched using 25% KOH solution at 40 °C. As the amorphous Si lacks any crystalline order, the etching by KOH is isotropic and also etches sideways. The geometry of the undercut features was dependent on the duration of this isotropic etch process and was varied from 2–10 min.

Alternatively, also a reactive ion etching (RIE) process was developed to avoid wet chemical etching. An RF power setting of 30 W with a medium chamber pressure of 10 mTorr, a gas flow of 20 sccm SF_6_ and 10 sccm Ar was used. The etch rate in Si was at least one magnitude faster than in the PTM-layer.

### 2.2. Multilayer Undercut Masters

This work was motivated by the wing surface of the Morpho butterfly, which is a periodic, multilayered ridge on the scales of the butterfly’s wings [1,38]. To achieve multilayer undercut structures to resemble those found in the Morpho butterflies, the fabrication approach was adopted in three ways:

Firstly, as an etch mask, a lithographically patterned gold layer was used instead of the laser-patterned PTM layer. For lithography either (i) optical photolithography, (ii) electron beam lithography with 350 nm broad features, or (iii) nanoimprint lithography with 300 nm features were employed. For photolithography, AZ5214E (Microchemicals, Ulm, Germany) was used in image reversal mode to generate 1.6 µm high resist patterns for the subsequent lift-off process. For electron beam lithography the PMMA-resist 950K A4 with a thickness of 220 nm was used, and the exposure of lines with a width of 350 nm was performed with a Raith e-line system at 10 kV and a beam current of 200 pA. With electron beam lithography nanostructuring of larger areas requires inefficiently long exposure time. As an alternative for the nanopatterning of larger areas exceeding 1 cm² NIL has proven to be a faster process [39]. For the NIL-made etch template a stamp with a checkerboard-style array of 300 × 300 nm^2^ mesas was used to imprint these square structures into a NIL material layer. For fabrication of a metal nanomesh array a dual layer lift-off nanoimprint process using LOR1A (MicroChem Corp., Westborough, MA, USA) and mr-NIL212FC resist materials (*micro resist technology* GmbH, Berlin, Germany) was used as described in [40].

A gold layer with 200 nm nominal thickness was deposited on the lithographic pattern by sputtering with 25 W power using a VonArdenne (Dresden, Germany) LS320 Sputter deposition system or by thermal evaporation using a Balzer’s BAK600 (Oerlikon Balzers Coating AG, Brügg, Switzerland) thermal evaporation system. In both cases, a sub-10 nm Cr layer was deposited as an adhesion layer on the substrate. By lift-off, the metal hard mask for the further NIL master fabrication was obtained.

Secondly, instead of using a single Si layer, a stack of alternating Si-SiO_2_ layers was used, as sketched out in Figure 4b. The sputter deposited layer stack was comprised of 6 pairs of a 50 nm thick amorphous Si layer on top of an 85 nm thick SiO_2_ layer. For a multilayer structure, the individual Si-layers have to be separated by interspaces that can also be etched (to continue etching of the subsequently lower Si-layer). While the amorphous Si was the layer to be underetched sideways, the interspace layer should display a significantly lower etch rate sideways.

Therefore, thirdly, instead of the pure wet chemical etching a reactive ion etching (RIE) process was used. Dry etching was performed in an Oxford Instruments (Oxford Instruments Plasma Technology, Yatton, UK) Plasmalab 100 system using the process gases SF_6_, O_2_, and Ar. The process pressure was varied between 5 and 60 mTorr to obtain optimal etching results. Si and SiO_2_ can both be dry etched by reactive ion etching (RIE) with a fluorine species, but the etch rate of SiO_2_ is significantly smaller. The PTM layer (as used in the previous section) is not a suitable mask layer for RIE, as it can only be wet etched after phase transition by blue light exposure.

The RIE process was optimized as a sequence of two etch steps: The first etch step was designed to etch anisotropically through the Si/SiO_2_-stack. Hence, this first step was performed at 40 W RF power with a low chamber pressure (5 mTorr), for a higher bias voltage and longer mean free path length. Additionally, to quench the etch selectivity of Si over SiO_2_ during the anisotropic etch phase, also oxygen was added to the fluorine etchant (5 sccm SF_6_, 24 sccm O_2_, 1 sccm Ar). The oxidation of the surface of the Si-layer resulted in quenching the otherwise higher etch rate of Si in contrast to SiO_2_ and avoided a side etch into the Si during the anisotropic etch phase. Process step duration for anisotropic etching was in the range of 20 min to 60 min depending on the number of layers. In the second etch step (40 W RF), the underetching was performed by using a strongly isotropic etch process. In this second step, more fluorine and less oxygen was added (20 sccm SF_6_, 1 sccm O_2_, 9 sccm Ar), and the etch process with the F-species was performed at higher chamber pressure (30 mTorr). Tuned in such a way, this RIE process achieved the under-etching into the Si-layers within 30 s. The resulting master stamp structures were inspected by scanning electron microscopy (SEM) (LEO 1530 VP, ZEISS Microscopy Jena, Germany). To recognize and to measure the undercut either SEM imaging with a tilted view angle or of a cross-section through the undercut structure was performed. Cross-sections were prepared by ion milling with a Zeiss Neon 40 EsB focused ion beam system (ZEISS Microscopy, Jena, Germany).

## 3. Results

The single-layer T-shaped masters demonstrate the feasibility to fabricate undercut structures also on a large scale. The PTM hard mask [41] was manufactured by Blu-Ray mastering, which is an industrial production technique used for optical memory devices of 120 mm diameter. For these Blu-Ray discs also industrial fabrication equipment exists and fabrication of large quantities has been proven. Using the same industrial fabrication environment for NIL masters opens a quickly accessible supply route for NIL masters at low costs. Figure 1 shows the successful realization of a NIL master with a single layer undercut structure.

As test structures, four squares of 25 × 25 mm were patterned (see Figure 1c). The Blu-Ray-technology-based patterning yielded sub-µm structures that serve as an etch mask and subsequently as the suspended lamellae. Etching of the exposed PTM material revealed a line edge roughness in the range of 20 nm. This is assumedly due to the spacing between the writing tracks, which leads to a curved front of phase transition. For exposure, the orthogonal pattern of our master had to be translated into exposure intervals on the spiral-shaped tracks that the laser was writing on the rotating substrate.

**Figure 2:**
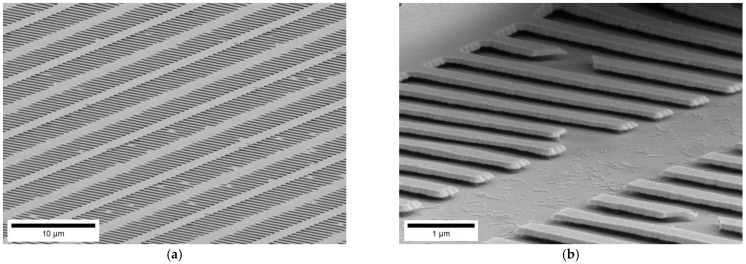
Defects on master after demolding from the NIL-substrate. (**a**) Overview image and (**b**) close-up of defect on master.

The patterned PTM layer was used as a hard mask to etch the aSi-layer in a wet isotropic KOH etch. As shown in the FIB-cross-section in Figure 1a the sideways underetch was in the range of 150 nm. These single-layer undercut structures were successfully produced over an area of several cm^2^ (see Figure 2a). During etching of the Si-layer also the line edge roughness from the PTM layer is transferred into the Si-layer. The FIB-cross section of the underetched Si layer also shows that the distance of underetch is often not identical on the left and the right side (Figure 1a). With wet chemical etching, the diffusion in the underetched cavities is critical, and concentration gradient may lead to inhomogeneous etch rates on opposing sides of the same structure. Despite this restriction, the overall structure fidelity was good over large areas (Figure 1b). Preliminary experiments also demonstrated the feasibility to replicate the inverse structure by NIL. During demolding, the master remained widely intact, which indicates the excellent adhesion of the PTM-layer on the Si-layer (Figure 2a). Damage to the master structures was only evident on few positions throughout the substrate.

**Figure 3:**
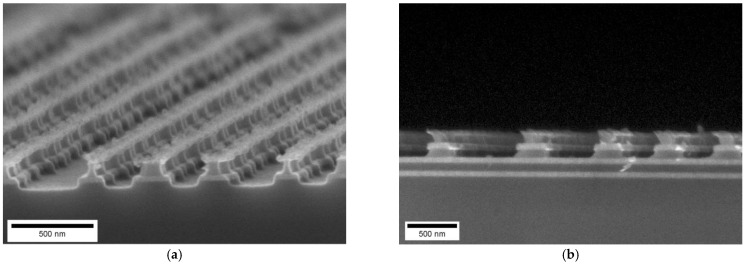
A PTM-Si-stack is etched by a F-based RIE process. (**a**) single layer PTM/Si-stack and (**b**) quadruple layer PTM/Si-stack. The cross-sections were fabricated by cleaving the wafer. Images were recorded by SEM on a vertically mounted master chip.

For the single layer undercut structure the PTM layer was both (a) laser-patterned hard mask on top and (b) etch stop layer below the Si-layer, which was to be underetched by KOH. Alternatively, also a RIE-process was investigated to avoid the complications of wet chemical etching. Figure 3a shows the same structure as Figure 1 only that the underetching was performed by dry etching. It can be seen in Figure 3a that also the PTM etch stop layer for KOH has been etched, so that the etching continued into the Si substrate. For the T-structure, it is evident, that the top PTM-structure was diminished from an initial 50 nm thickness to a thickness below 20 nm. The result was taken as stimulus to also use a quadruple stack of PTM-layer on top of a Si-layer. Etching such a multi-stack allowed to fabricate a dual layer undercut master and to demonstrate the feasibility to produce more complex undercut structures. However, the fluorine-based RIE process also etches the PTM hard mask on top (Figure 3b).

As PTM is both, the pattern defining hard mask and the interlayer between aSi-layers, the RIE process equally removes the PTM interlayer and the pattern-defining hard mask. We conclude that with the given material stack and the single-step RIE process no complex multilevel underetched structures are feasible.

**Figure 4:**
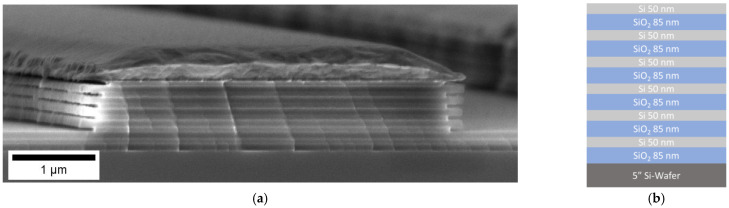
(**a**) A multilevel underetched structure with a gold mask defined by optical lithography. The lithographic structure is 5 µm wide. SEM image on the cross-section of the layer stack after the dual-stage etching (first etch step stopped after Si layer #5). (**b**) Schematic sequence of layers (without the gold hard mask).

For this reason, we progressed to a different material system—aSi and SiO_2_—for the multilayer undercut masters. Due to a size incompatibility of the PTM sputter systems with the SiO_2_ sputter system, patterning by Blu-Ray mastering had to be replaced by conventional lithography. As hard mask material, a gold layer was used. The hard mask for the sample shown in Figure 4a was a gold layer prepared by optical lithography and lift off. The multi-stack of Si and SiO_2_ is schematically illustrated in Figure 4b. The etching process was optimized by tuning the pressure and the mixture ratio of the etch gases SF_6_, O_2_, and Ar. By changing the gas composition, the pressure and the inductive power, a change from anisotropic to isotropic etching could be achieved. In the first RIE etch step the layer stack was anisotropically etched, while in a second etching step the Si-layers were isotropically etched sideways, while the SiO_2_ interspace layers were not etched significantly. This resulted in the multilevel underetched structure shown in Figure 4. The hard mask for the sample shown in Figure 4a was a gold layer prepared by optical lithography and lift-off. Even after etching the gold layer is still present. This gives rise to the expectation, that even thicker layers or more layers could be processed by the same process. The high anisotropy of the first RIE step can be recognized by the vertical escape line of the corners of the SiO_2_ layers in Figure 4a. The underetch into the Si-layer was in the range of 300 nm in top Si-layers down to 150 nm in bottom Si-layers. This gradually decreasing underetch width is analogous to the wing structure on Morpho butterfly. The wing structures are not in the µm range but the sub-µm range. Hence, smaller patterns with feature sizes in the 500 nm to 100 nm range were fabricated.

**Figure 5:**
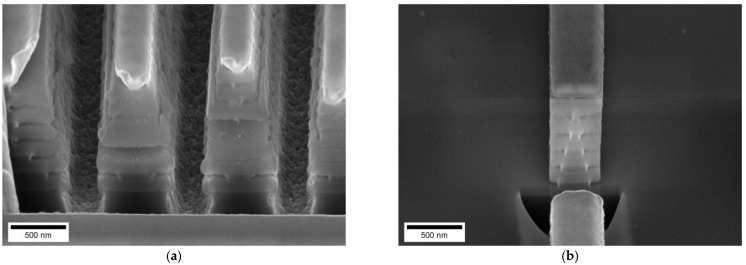
A multilevel underetched structure with a gold hard mask defined by electron beam lithography. (**a**) SEM image on the cross-section of the layer stack after etching. (**a**) Densely packed lines with little spacing. Lithographic structures are 400 nm (middle-left) and 350 nm (middle-right) wide. (**b**) Line with more than 1 µm inter-line-distance. The lithographic structure is 350 nm wide.

To achieve these sub-µm structures we used either electron beam lithography or nanoimprint lithography to prepare the hard mask pattern. An example obtained using e-beam lithography is shown in Figure 5. The sideways underetch distance of Si was in the range of 120 to 200 nm. The remaining ridge of Si was already thin and reached the limit of mechanical stability. By shortening the duration of the second RIE etch step, also less undercut can be achieved. For a densely packed pattern of lines and spaces (Figure 5a) the anisotropic etch process did not yield a perfectly vertical envelope of the SiO_2_ lamellae. Increasing the spacing between lines (Figure 5b) yielded vertically aligned corners of the SiO_2_ lamellae. Also, here the amount of the sideways underetch into the Si layers decreased from top (more underetch) to bottom (less underetch). While the fabrication of multilevel undercut structures was demonstrated to be feasible also in the nm-range—a range that is relevant for biomimetic structures—the method of electron beam lithography is not suitable for patterning on large areas.

**Figure 6:**
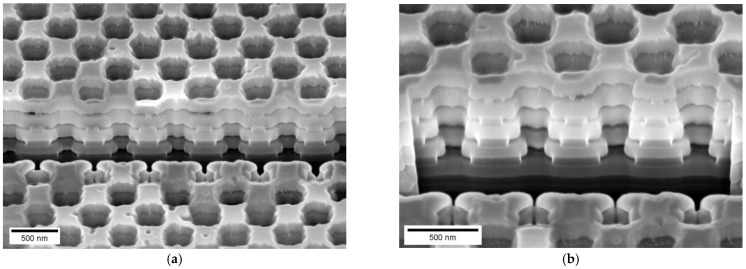
A multilevel underetched structure with a gold hard mask was defined by a NIL imprinted stamp. A 2D checkerboard structure consisting of 300 × 300 nm^2^ squares was transferred into a metal layer acting as etch hardmask. The checkerboard pattern was transferred into the material by RIE etching. (**a**) Overview; (**b**) close-up.

In a final experiment, we developed a process for larger area patterning of multilevel undercut structures. The industrial NIL replication using NIL-steppers and NIL roll-to-roll systems is excellently suited to fabricate 3D-structures on large areas. In this approach, two-dimensional nanoimprint lithography was used to prepare the master for three-dimensional nanoimprint lithography. A NIL stamp with 300 nm features was used to fabricate a gold hard mask analogous to the previous experiments (see Figure 4 and Figure 5). By a two-step RIE procedure this two-dimensional checkerboard pattern of the gold hardmask was transferred into a three-dimensional structure of the multilevel undercut master. Concluding, a three-dimensional NIL master was fabricated using a two-dimensional NIL-patterned hard mask by etching and is shown in Figure 6.

The FIB-milled cross-section reveals four layers of undercut structures. Even with these 300 nm structures, a clear undercut could be achieved. For the top Si layer, the width of the remaining Si is below 50 nm, which is already at the limit of the mechanical stability of the master. Similar to the Morpho butterfly wing structure, the structures get wider from top to bottom. With 300 nm features the resolution required for biomimicry of the blue butterfly wing of Morpho menelaus could be achieved. The repeated replication of NIL masters with multilayer undercut T-structures has already been demonstrated in [42] and marks an important step towards the reproducible fabrication of biomimetic nanostructures. With the availability of a production procedure for multilevel undercut master structures, the first step towards the technological use of NIL for structural colors has been made.

## 4. Conclusions

Nanoimprint lithography is an exceptional patterning technique, as it can directly replicate three-dimensional structures. The availability of a three-dimensional master is a bottleneck of the NIL replication. In this work, we have presented fabrication technologies for undercut three-dimensional masters. With the structural design of our masters, we were inspired by the structural color of Morpho butterfly wings.

We have successfully demonstrated different ways to fabricate undercut nanoscale features on large areas. In a first step, a two-dimensional hard mask was prepared on a multilayer stack. In a subsequent two-stage RIE-process the undercut features were fabricated. The use of PTM hard masks allows using the industrially established method of laser writing as used for Blu-Ray mastering. Alternatively, also optical lithography, electron beam lithography and even nanoimprint lithography itself have been successfully demonstrated as alternative approaches to fabricate hard mask layers. With the industrially preferred RIE process, the problem-prone wet etching can be avoided. With the two-stage RIE process an industrially viable route to produce multilevel undercut structures was demonstrated. With an optimized process we could successfully show the fabrication of a four-layer undercut structure with 300 × 300 nm features.

In future generations these approaches—PTM hard mask fabrication by Blu-Ray mastering and multilayer undercut etching by dual-stage RIE—may be combined. This will yield an industrially relevant fabrication approach for complex three-dimensional masters. The multilevel undercut master structures produced in this work are an essential component to further develop an imprinting and demolding procedure for complex undercut structures. NIL steppers and NIL roll-to-roll systems may transfer this approach to the industrial fabrication of structural colors on large areas.

## Figures and Tables

**Table 1:** Overview of five process sequences used to fabricate undercut T-structures

T-Structure	Single-Layer		Multi-Layer		
Patterning	Blu-Ray laser		g-line lithography	e-beam lithography	NIL
Lift-off mask	-		AZ5214E	PMMA	mr-NIL212
Etch-mask	Inorganic PTM-layer		Au-hardmask		
Etch Step 1	KOH wet etch	Medium pressure RIE	Low pressure RIE		
Etch Step 2			High pressure RIE		

## Data Availability

The data presented in this study are available on request from the corresponding author after agreeing to non-disclosure. The raw data are not publicly available due to non-disclosure agreements within the grant consortium.
